# Maternal health in resource-poor urban settings: how does women's autonomy influence the utilization of obstetric care services?

**DOI:** 10.1186/1742-4755-6-9

**Published:** 2009-06-16

**Authors:** Jean-Christophe Fotso, Alex C Ezeh, Hildah Essendi

**Affiliations:** 1 African Population and Health Research Center (APHRC), Nairobi, Kenya

## Abstract

**Background:**

Despite various international efforts initiated to improve maternal health, more than half a million women worldwide die each year as a result of complications arising from pregnancy and childbirth. This research was guided by the following questions: 1) How does women's autonomy influence the choice of place of delivery in resource-poor urban settings? 2) Does its effect vary by household wealth? and 3) To what extent does women's autonomy mediate the relationship between women's education and use of health facility for delivery?

**Methods:**

The data used is from a maternal health study carried out in the slums of Nairobi, Kenya. A total of 1,927 women (out of 2,482) who had a pregnancy outcome in 2004–2005 were selected and interviewed. Seventeen variable items on autonomy were used to construct women's decision-making, freedom of movement, and overall autonomy. Further, all health facilities serving the study population were assessed with regard to the number, training and competency of obstetric staff; services offered; physical infrastructure; and availability, adequacy and functional status of supplies and other essential equipment for safe delivery, among others. A total of 25 facilities were surveyed.

**Results:**

While household wealth, education and demographic and health covariates had strong relationships with place of delivery, the effects of women's overall autonomy, decision-making and freedom of movement were rather weak. Among middle to least poor households, all three measures of women's autonomy were associated with place of delivery, and in the expected direction; whereas among the poorest women, they were strong and counter-intuitive. Finally, the study showed that autonomy may not be a major mediator of the link between education and use of health services for delivery.

**Conclusion:**

The paper argues in favor of broad actions to increase women's autonomy both as an end and as a means to facilitate improved reproductive health outcomes. It also supports the call for more appropriate data that could further support this line of action. It highlights the need for efforts to improve households' livelihoods and increase girls' schooling to alter perceptions of the value of skilled maternal health care.

## Background

Despite various national and international initiatives to improve maternal health, more than half a million women from developing countries die each year as a result of complications related to pregnancy and child birth [1,2]. With approximately 247,000 maternal deaths per year, sub-Saharan Africa shares nearly half of the toll despite accounting for less than 12 percent of the world population [3]. There is wide recognition that one major factor contributing to high maternal mortality is the low use of maternal health services for delivery; yet the proportion of deliveries assisted by health professionals in sub-Saharan Africa has remained very low and progressed only marginally from 42 percent in 1990 to 46 percent in 2004 [4]. Focusing on the period around childbirth is appropriate since most maternal deaths cluster around labor and the postpartum period. In recognition of the central role of professional care at birth, skilled birth attendance was chosen as a process indicator for monitoring progress towards Millennium Development Goal (MDG) number 5 that seeks to reduce maternal mortality by three-quarters by 2015.

In seeking to explain these low levels of health care utilization in developing countries, most studies have focused on provision and geographic accessibility of services, and relatively very few have looked at how factors such as women's autonomy influence use of services [5,6]. The 1994 International Conference on Population and Development Programme of Action noted that "improving the status of women also enhances their decision-making capacity at all levels in all spheres of life, especially in the area of sexuality and reproduction [7]". A woman's autonomy is generally defined as the ability to make and execute decisions regarding personal matters of importance on the basis of the woman's power over others, access to information, control over material resources, and freedom from violence by her husband or other men [8-10]. Others have conceptualized autonomy as women's ability to determine events in their lives, even though men and other women may be opposed to their wishes [11,12]. Items widely explored include women's freedom of movement, discretion over earned income, decision making related to economic matters, freedom from violence or intimidation by husbands, and decision making related to health care [11-14].

A number of studies have examined the effect of women's autonomy and their reproductive health outcomes in the context of the developing world. Unfortunately, this literature has been growing asymmetrically, the body of knowledge being built mainly on evidence from Asia and South Asia in particular [12-19], with very little empirical evidence from sub-Saharan Africa where the patterns of women's status and social position have been shown to differ from those observed in Asia and other parts of the developing world [11,20]. These studies have shown that lower family size and desired fertility are observed among women with higher levels of autonomy [20]; higher rates of contraceptive prevalence were recorded among women with greater interpersonal control [17,18]; and lower rates of child mortality were observed among women with more decision-making power [14]. Comparatively much less research has focused on the relationship between women's status and the use of health services, a proximate determinant of maternal and child mortality [8,14].

Another research gap that can be identified from previous research is the construction of women's autonomy. While most researchers agree that the impact of women's autonomy on demographic and health outcomes should be investigated using a combination of measures reflecting women's degree of control in their lives, including particularly control over financial resources, decision-making power, and the extent of freedom of movement [8,13,14,17-19], the context and reasons for using a single measure or a group of indicators reflecting different aspects of autonomy, has not been fully clarified. This paper agrees with Saleem & Bobak [19] and argues that both approaches should be used simultaneously. Noticeably, most of the previous studies on the extent to which women's autonomy influences their demographic and reproductive health outcomes have used models that failed to appropriately control for household wealth. For example, Saleem & Bobak [19] used four indicators of wealth: water supply, house construction, toilet facility, and husband work status. Not only are these indicators likely to be highly correlated; the absence in the model of more wealth-related indicators such as household possessions may result in misestimating the effect of autonomy. Besides filling some of the substantive and methodological research gaps above-presented, this piece of work focuses on the urban poor.

### Why lay focus on the urban poor?

In sub-Saharan Africa, the unprecedented population growth that started in the second half of the 20^th ^century has evolved into unparalleled urban growth. The region's urban population was 15 percent in 1950, 32 percent in 1990, and the United Nations projects that by 2030, a majority of sub-Saharan Africa's population will live in urban areas [21]. The essential feature of current African urbanization is that the pace of urbanization has outstripped economic growth, making it difficult for national and urban authorities to provide affordable housing, quality social services or sufficient employment to the growing population [22]. These trends have resulted in unprecedented growth of slums and unplanned settlements on the periphery of most African cities.

Kenya's capital city typifies the current urban population boom and the associated urban health and poverty problems. Its population increased from about 120,000 in 1980 to about 3 million in 2000, with over 60% of the population living in slums which cover only 5% of city's residential land area [23]. It is estimated that while absolute poverty increased from 48 to 53% in rural areas of Kenya between 1992 and 1997, it almost doubled from 27 to 50% over the same period in Nairobi city [24]. Key dimensions of poverty include inadequate access of urban dwellers to appropriate health care services, with Nairobi slums being served mainly by privately-owned, sub-standard, unlicensed and informal health facilities [25]. Young people in these informal settlements face challenges such as high levels of unemployment, crime and substance abuse, poor schooling facilities, and early sexual debut resulting in unplanned childbearing which accounts for a substantial proportion of births in Kenya [26].

The advantage that urban areas previously had over rural areas on various health, social and economic indicators has narrowed over time, as economic and environmental conditions have sharply deteriorated in rapidly growing cities [27,28]. It is increasingly evident that failing to appropriately target the growing sub-group of the urban poor and improve their living conditions and health status – which is an MDG target itself-may result in lack of improvement on national indicators of health and consequently, push countries away from meeting the MDG targets.

The research presented in this paper examines possible influences of the extent of women's autonomy on their use of maternity services. It was guided by the following questions: 1) How does women's autonomy influence the choice of place of delivery in resource-poor urban settings? 2) Does its effect vary by household wealth? and 3) To what extent does women's autonomy mediate the relationship between women's education and use of health facility for delivery?

## Methods

### Data

The data used in this paper are from a maternal health study carried out in 2006 in two slums of Nairobi, Kenya, namely Korogocho and Viwandani [29]. The two communities exhibit structural differences: Viwandani is home to many industrial workers; it attracts migrants with relatively higher educational levels, and exhibits higher levels of economic activity; whereas the population in Korogocho is more stable. Using the Nairobi Urban Health and Demographic Surveillance System (NUHDSS), all women aged between 12 and 54 years who lived in the study area and had a pregnancy outcome between January 2004 and December 2005, were selected and interviewed between. A total of 2,482 women were identified as eligible for the survey, and a total of 1,927 interviews were successfully conducted. The 555 missing women had either out-migrated from the study area or were absent during the survey. Written informed consent was read to respondents before the interview. Further, all health facilities serving the study population were assessed with regard to the number, training and competency of obstetric staff; services offered; physical infrastructure; and availability, adequacy and functional status of supplies and other essential equipment for safe delivery, among others. A total of 25 facilities were surveyed. Ethical approval for the study was sought from the Kenya Medical Research Institute.

### Dependent variable

The outcome variable is place of delivery. From the health facility survey and borrowing from other work [30], health facilities were classified as either appropriate or inappropriate. The first group, labeled as "*inappropriate*", comprised 17 small and often ramshackle and unlicensed clinics and maternity homes that were deemed unable to offer many of the signal functions of Basic Emergency Obstetric Care (BEOC). They are located within the two slum communities. The second category comprised eight facilities that provide at least basic essential obstetric care. These facilities are run or owned by government, religious and missionary groups or Faith-Based Organizations (FBOs), and large Non-Governmental Organizations (NGOs), and are located in the outskirts of the slums or other places in the city, often far from the slums. Health facilities in this category were labeled as "*appropriate*". Based on this grouping, the dependent variable is defined as follows:



### Women's autonomy

The commonly used dimensions of women's autonomy include women's freedom of movement; discretion over earned income; decision making related to economic matters; freedom from violence or intimidation by husbands; and decision making related to health care [11-14,19]. The maternal health study that generated the data used for this paper only collected variables that relate to women's decision-making autonomy and freedom of movement (see Additional file 1). For the purpose of this study, we define an overall women's autonomy variable using all items, as well as decision-making autonomy (from the 10 variables) and freedom of movement (based on seven remaining item variables). All three autonomy variables are constructed using Principal Component Analysis (PCA) and recoded as tertiles with categories labeled low autonomy, middle autonomy and high autonomy.

### Control variables

The socioeconomic variable of interest to this study is household wealth. PCA was used to generate household wealth tertiles from household characteristics, namely, the presence of electricity, type of cooking fuel, material of the dwelling floor, source of drinking water, type of toilet facility, and possession of bed net, solar, radio, television, refrigerator, bicycle, motorcycle/scooter, car/truck and mobile phone. The categories of the constructed variable were labeled poorest, middle and least poor. Women's education (coded as none; primary; and secondary or higher) was also included. Other control variables were parity, age at delivery, pregnancy wantedness, number of antenatal visits, advice during antenatal care to deliver with a skilled health care provider, and slum residence (Korogocho, Viwandani).

### Methods of analysis

To achieve its objectives, the paper fitted ordered logistic regressions using partial proportional odds models [31]. The analysis is carried out in three phases. First, multivariate models are used to identify factors associated with place of delivery and quantify their net effects. Second, interaction models are examined to test the extent to which the effects of women's autonomy on choice of place of delivery vary by household wealth. Third, we examine the potential mediating effect of women's autonomy on the link between education and place of delivery by adjusting the effect of education for autonomy and assessing the change in the coefficients. At each stage, we run a model with women's overall autonomy and another with both women's decision-making and freedom of movement.

## Results

### Sample description

Additional file 2 depicts the description of the sample of 1,927 women who were interviewed in the household survey. About two-thirds of the women had primary education, and only one-quarter reached or went beyond secondary education. Nearly 31% of the respondents reported that their pregnancy was either mistimed or unwanted. Only 52% of the women made the recommended four antenatal care (ANC) visits, and during the ANC visits, about 77% of the women were advised to deliver with the help of a health professional. For a quarter of the women, it was their first pregnancy; about 46 percent had two or three children, while the remaining 29 percent had four or more children. Finally, 57 percent of the respondents were from Korogocho and 43 percent from Viwandani.

### Multivariate analysis: Main effects

As can be seen in Additional file 3, while the effect of household wealth on the choice of place of delivery appeared to be strong and in the expected direction (p < 0.01 in both Panel A and B), the effect of women's overall autonomy was insignificant and counter-intuitive, with low autonomy women the least likely to deliver in appropriate health facilities (see Panel A). Panel B shows that neither decision-making autonomy nor women's freedom of movement had any influence on place of delivery.

Women with at least secondary education were more likely to deliver in a health facility in general or in an appropriate health facility, compared to those with no education (p < 0.01). Pregnancies that were wanted were more likely to be delivered at health facilities (p < 0.01 in Panels A and B) or at appropriate facilities (p < 0.01 in Panel A), compared with those that were either mistimed or unwanted. The number of antenatal visits was associated with place of delivery; women who made the recommended four visits were more likely to deliver in a health facility in general (p < 0.01) or in an "appropriate" health facility (p < 0.01), compared to their counterparts who made one or no visits. Importantly, respondents who were advised during antenatal care to deliver at a health facility were significantly more likely to use health facilities in general (p < 0.01 in both Panels) and to use the well-equipped ones in particular (p < 0.01 in both Panels), compared to those who were not advised. The likelihood of delivering at a health facility in general and in the well-equipped facilities in particular, significantly decreases as parity increases (p < 0.01 in both Panels). Women aged less than 25 years were the least likely to deliver at health facilities or at the appropriate ones. Compared to Viwandani residents, Korogocho women were twice as likely to deliver at a health facility in general (p < 0.01), and more than five times as likely to do so in an appropriate facility (p < 0.01).

### Multivariate analysis: Interaction effects

**Figure 1:**
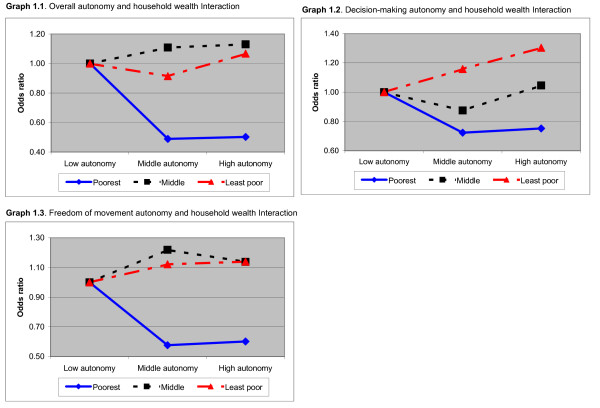
**Interactions between women's autonomy and household wealth as determinants of health facility delivery**. Graph 1.1. Overall autonomy and household wealth Interaction. Graph 1.2. Decision-making autonomy and household wealth Interaction.

Results of the interactions between women's autonomy and household wealth are summarized in Additional file 4, with focus on delivery at appropriate health facilities. Detailed results of the interactive model are shown in Additional file 5. Noticeably, the effects of women's autonomy vary greatly by household wealth; in particular, the counter-intuitive result in the multivariate-main effects model is only observed among the poorest. Graph 1-1 of Figure 1 indicates that among middle and least poor households, high overall autonomy women were slightly more likely to deliver in appropriate health facilities, compared with their low or middle autonomy counterparts. Though middle autonomy women in least poor households tended to exhibit lower use of appropriate health facilities, the pattern of association between women's overall autonomy and place of delivery among women residents in middle and least poor households is in line with expectation. Similar patterns are observed for decision-making autonomy (Graphs 1–2) and freedom of movement autonomy (Graph 1–3), with graded relationships between decision-making or freedom of movement and place of delivery viewed among women from least poor households.

### Does women's autonomy mediate the effect of education?

We examine the potentially mediating effect of autonomy on the association between women's education and choice of place of delivery, by adjusting the effect of education for autonomy and assessing the changes in the coefficients. As can be seen in Additional file 6, the changes between the model without autonomy variables and the model with autonomy variables were modest, especially for secondary education. This minimal change suggests that autonomy may not be a major mediator of the link between education and use of health services for delivery.

## Discussion

It has been hypothesized that higher autonomy of women would translate into improved health seeking behavior and consequently, into better health outcomes [6]. However, the results of this study show that in the Nairobi informal settlements, utilization of maternal health services for delivery is not enhanced by high levels of women's overall autonomy, freedom of movement, or decision making; a finding consistent with other studies that have examined the influence of women's autonomy on various health outcomes. Using data from the demographic and health surveys, Dodoo's findings [32] provide no support for an association between women's autonomy and decisions regarding child health in Ghana. A study in Nepal also found that the influences of women's involvement in decision-making regarding their own health or large purchases on antenatal care attendance or skilled delivery care, were rather weak, while discussion of family planning between spouses was linked to increased likelihood of receiving skilled antenatal and delivery care [6]. Similarly, weak relations emerged between women's autonomy – measured by decision-making and freedom of movement as we do in this paper – and the use of family planning in Oman [15]; and no consistent relationship was found between low autonomy and child mortality among Muslims and non-Muslims in India, Malaysia, the Philippines and Thailand [13].

A number of other studies revealed mixed conclusions on the effect of women's autonomy on various aspects of maternal health. While women's freedom of movement appeared to be a major determinant of maternal health care utilization among poor to middle-income women in a large urban area of Uttar Pradesh, the two other dimensions investigated (control over finances and decision-making) were not significantly associated with the outcomes studied [12]. Similarly, Saleem and Bobak [19] showed that decision-making autonomy was associated with contraception while movement autonomy was not.

However, a number of other studies have clearly demonstrated that women's autonomy has a strong and consistent effect on reproductive health outcomes. Bloom et al. [12] demonstrated that women's autonomy was a major determinant of maternal health care utilization among urban poor to middle-income women in a North Indian city. The study used a two-face cluster design concentrated on households within a 15-minutes walking distance to a government or charity facility where health care is given free of charge. The proximity to health services and the fact that care was provided free of charge, may suggest that women's autonomy is a major enhancer of maternal health in a context of higher geographic accessibility and lower minimal financial costs. This view that women's autonomy may only be relevant in conducive environments seems to agree with Mason who noted that women's autonomy, coupled with education and communication with spouses greatly influences use of contraception [20]. This interpretation is only based on speculations, and requires additional, in-depth research for support. It is worth noting that studies that have shown strong linkages between women's autonomy and maternal health outcomes have been conducted in Asia, a region with cultures that are different from sub-Saharan Africa's, especially regarding kinship structures, with possible bearing on the way women's autonomy plays a role on reproductive health. It should also be mentioned that very few studies have focused on informal settlements which may have specific conditions such as lack of social networks and cohesion among its residents.

It is also important to note that not only is the influence of women's autonomy not statistically significant in the research presented in this paper, the negative sign observed in some of the coefficients in Additional file 3 suggests that the results tend to run contrary to the expectation emanating from the hypothesis that maternal health outcomes are hampered by low levels of women's autonomy. Before we dismiss the important role of women's autonomy on maternal health services utilization, and in line with the interpretation mentioned above, we examine the interaction between women's autonomy and household wealth as determinants of maternal services utilization. Our results clearly show that women's autonomy is an enhancer of maternal health service utilization among the middle-income and least poor groups in the slums of Nairobi.

Strikingly, this study shows that among the poorest, lower autonomy women tend to exhibit higher use of maternity services, a finding which is contrary to expectation. Other authors have contemplated the possibility that the relative status of women may be inversely associated with health outcomes [32] or have speculated that the formulation of the questions related to women's autonomy may offer at least a partial explanation of the counter intuitive results [6]. One could argue that poorest women, because they are likely to be uneducated, may have a different understanding of autonomy-related issues.

The multivariate analyses confirm the well known effects of key socio-demographic characteristics on the utilization of maternity services. The most notable effects are those of education and household wealth, as shown by various other authors [26,30,33]. Compared to Viwandani residents, Korogocho women were about 50% as likely to deliver at a health facility in general, and more than four times as likely to do so in an appropriate one. This could be explained by the fact that appropriate facilities in Viwandani are limited and a higher number of the respondents (57%) in this study are from Korogocho which is better served with appropriate health facilities compared to Viwandani. Also, consistent with other studies, women with higher parity were less likely to deliver in well-equipped health facilities; women aged less than 25 years were the least likely to deliver at health facilities or at the appropriate ones, a finding consistent with a study which found that teenagers in sub-Saharan Africa experience poorer maternal health care than older women with similar characteristics [34].

There is a strong association between the use of antenatal care services and delivery at a health facility. Interestingly, women who were advised during antenatal visits to deliver at a health facility were more likely to do so. This highlights the need for poor urban women to have such maternal health services as contraception, delivery and post-partum care alongside other health promotion messages to improve their health-seeking behavior. Access to family planning services is also crucial in improving delivery care. About 30% of the women in this study reported that they did not intend to get pregnant with the current baby, and the multivariate results clearly indicate that unplanned pregnancies were less likely to be delivered at appropriate health facilities.

Very few studies have focused on autonomy being a mediator between education and health service utilization. Results from our study indicate very modest changes in coefficients between models with and without autonomy variables, indicating that while education is a major determinant of health seeking behavior, its effect is not mediated by women's autonomy. This result agrees with Saleem and Bobak [19] in their study on women's autonomy, education and contraception in Pakistan.

## Conclusion

The study has shown that the influence of women's autonomy on the utilization of maternal health services among poor women in Nairobi is weak on average. However, this conclusion masks strong differences between wealth groups: While household wealth, education and demographic and health covariates had strong relationships with place of delivery, the effects of women's overall autonomy, decision-making and freedom of movement were rather weak. Among middle and least poor households, all three measures of women's autonomy were associated with place of delivery, and in the expected direction; whereas among the poorest women, they were strong and counter-intuitive. Most researchers acknowledge the importance of women's autonomy, education and income as key predictors of health seeking behaviors in developing countries. Our findings suggest that the important question is not whether the three factors are important, but how they interplay to influence maternal health outcomes.

From a policy point of view, the paper does not offer any answer that could be accepted and adopted without further elaboration. But it certainly argues in favor of broad actions to increase women's autonomy both as an end and as a means to facilitate improved reproductive health outcomes [9]. In all, our findings support the call for more appropriate data that could further support this line of action. Most research on maternal health in the African context has focused on women-level determinants (e.g. education, income, age) or on factors that confront women with their external environments (e.g. infrastructure, access), neglecting the dominant effect of bargaining power within households or with male decision makers. It is reasonable to suggest that our understanding of the determinants of maternal and child health may be incomplete without more accurate measurement of power, decision-making processes and the paths through which they affect reproductive health outcomes [32]. Finally, the strong association of women's education and household wealth with utilization of maternal services highlights the need for efforts to improve households' livelihoods and increase girls' schooling to alter perceptions of the value of skilled maternal health care.

## Competing interests

The authors declare that they have no competing interests.

## Authors' contributions

JCF framed the research question, conducted the literature review, led in the data analysis, and contributed in the writing of the paper. ACE participated in the conception of the idea of this manuscript and provided guidance for the write up. HE participated in literature review and discussion of the results. All authors read and approved the final manuscript.

## Supplementary Material

Additional file 1 **Variables used to define women's autonomy**. The data provided represent the 17 variable items used to construct women's decision-making, freedom of movement, and overall autonomy. Click here for file

Additional file 2 **Characteristics of women from the slums of Nairobi, Kenya who delivered in 2004–2005**. The table shows details of the sample of 1,927 women used in the study. Click here for file

Additional file 3 **Coefficients of ordered logistic regression on the effects of women's autonomy on health facility delivery**. The data provided in this table shows the effects of the constructed women's autonomy on the choice of place of delivery. Click here for file

Additional file 4 **Interactions between women's autonomy and household wealth as determinants of health facility delivery**. This figure gives a summary of the interactions between the three measures of women's autonomy and household wealth in influencing women's place of delivery. Click here for file

Additional file 5 **Interactions between women's autonomy and household wealth as determinants of health facility delivery**. This table shows the coefficients of interactions terms between the three measures of women's autonomy and household wealth in influencing women's place of delivery. Click here for file

Additional file 6 **Changed in the coefficients of place of delivery by education after controlling for women's autonomy**. The table shows the results of the analysis of the potential mediating effect of women's autonomy on the link between education and place of delivery. Click here for file
