# Supplementary material for: Maternal health in resource-poor urban settings: how does women's autonomy influence the utilization of obstetric care services?
Source: Reprod Health. 2009 Jun 16;6:9. doi: 10.1186/1742-4755-6-9 (PMC2706794; doi:10.1186/1742-4755-6-9)
Supplement: Additional file 1 — Variables used to define women's autonomy. The data provided represent the 17 variable items used to construct women's decision-making, freedom of movement, and overall autonomy. [file 1742-4755-6-9-S1.doc]

**Table I**. Variables used to define women's autonomy

| **A. Decision making** |
| --- |
| 1. Who decides how the money you earn is spent?**^a^** |
| Who in your household usually has the final say on the following decisions**^a^** |
| 2. Your own health care? |
| 3. Making large household purchases? |
| 4. Making household purchases for daily needs? |
| 5. Visits to family or relatives? |
| 6. What food should be cooked each day? |
| 7. You should do work to earn money? |
| 8. What to do if a child falls sick? |
| 9. Having another child? |
| **B. Freedom of movement** |
| 1. If you are ill and need to see a doctor, do you first have to ask someone’s permission?**^b^** |
| Are you usually allowed to go to the following places on your own**^a^**: |
| 2. Just outside your house or compound? |
| 3. Local market to buy things? |
| 4. Local health center or doctor? |
| 5. In the neighborhood for recreation? |
| 6. Home of relatives or friends in the neighborhood? |

**^a^**Coded: 1=Respondent alone or with somebody else; 0=Other(s)

**^b^**Coded: 1=Yes; 0=No
